# c-Myb and C/EBPβ regulate OPN and other senescence-associated secretory phenotype factors

**DOI:** 10.18632/oncotarget.22940

**Published:** 2017-12-05

**Authors:** Kevin C. Flanagan, Elise Alspach, Ermira Pazolli, Shankar Parajuli, Qihao Ren, Laura L. Arthur, Roberto Tapia, Sheila A. Stewart

**Affiliations:** ^1^ Department of Cell Biology and Physiology, Washington University School of Medicine, St. Louis, MO, USA; ^2^ ICCE Institute, Washington University School of Medicine, St. Louis, MO, USA; ^3^ Department of Medicine, Washington University School of Medicine, St. Louis, MO, USA; ^4^ Siteman Cancer Center, Washington University School of Medicine, St. Louis, MO, USA

**Keywords:** senescence, SASP, osteopontin, c-Myb, C/EBPβ

## Abstract

Tumorigenesis results from the convergence of cell autonomous mutations and corresponding stromal changes that promote tumor cell growth. Senescent cells, which secrete a plethora of pro-tumorigenic factors termed the senescence-associated secretory phenotype (SASP), play an important role in tumor formation. Investigation into SASP regulation revealed that many but not all SASP factors are subject to NF-kB and p38MAPK regulation. However, many pro-tumorigenic SASP factors, including osteopontin (OPN), are not responsive to these canonical pathways leaving the regulation of these factors an open question. We report that the transcription factor c-Myb regulates OPN, IL-6, and IL-8 in addition to 57 other SASP factors. The regulation of OPN is direct as c-Myb binds to the OPN promoter in response to senescence. Further, OPN is also regulated by the known SASP regulator C/EBPβ. In response to senescence, the full-length activating C/EBPβ isoform LAP2 increases binding to the OPN, IL-6, and IL-8 promoters. The importance of both c-Myb and C/EBPβ is underscored by our finding that the depletion of either factor reduces the ability of senescent fibroblasts to promote the growth of preneoplastic epithelial cells.

## INTRODUCTION

Age is a major risk factor in the development of cancer [1]. In addition to the accumulation of epithelial cell mutations, age-dependent changes in the stromal compartment play an important role in tumor promotion [2–7]. One of these changes is the accumulation of senescent stromal cells that possess the ability to stimulate preneoplastic and neoplastic cell growth. First described as an *in vitro* phenomenon caused by repeated cell divisions, senescence can also be caused by a number of genotoxic stresses including telomere shortening or dysfunction, DNA double strand breaks, oxidative stress, tumor suppressor expression, and oncogene activation [8–10]. Senescent cells are associated with a flattened morphology, the presence of heterochromatic foci (SAHFs), positive senescence-associated β-galactosidase staining, and an altered gene expression and secretion profile termed the senescence-associated secretory phenotype [SASP; 11–14]. Significantly, senescence is now known to occur both *in vitro* and *in vivo* [9, 11, 15–18] where it impacts a diverse number of biologic processes including cancer.

Senescence acts as a potent tumor suppressive mechanism in a cell autonomous setting by preventing the proliferation of cells with activated oncogenes or excessive DNA damage. However, as individuals age, senescent cells accumulate within tissues where they are postulated to contribute to aging phenotypes [11, 12]. Aged mice cleared of p16^Ink4a^-positive senescent cells have reduced incidences of several age-related pathologies, including reduced tumor rates [2, 19]. Further, senolytic drugs that target senescent cells can ameliorate many age-related maladies, underscoring the importance of these cells in age related diseases [20–22]. Additionally, the largest risk factor for cancer is age, and there is significant evidence that accumulating senescent cells paradoxically contribute to cancer development and progression in a cell non-autonomous fashion. As with other age-related diseases, elimination of senescent cells reduces spontaneous tumor rates in naturally aged mice [19, 22]. The SASP can promote growth and transformation of epithelial cells in numerous models, suggesting that secretion of the SASP by accumulating senescent cells may contribute to age-related tumorigenesis [3, 4, 7, 13, 14, 23–25].

The SASP consists of numerous secreted factors including cytokines, mitogens, and extracellular matrix remodelers that are upregulated at the mRNA and protein levels [7, 14]. The regulation of SASP expression is complex and incompletely understood, but recent work has revealed that both the cell type and senescence inducer can significantly impact the mechanisms that regulate SASP expression as well as the specific SASP factors expressed [26, 27]. The expression of many factors, including the canonical SASP factors IL-6 and IL-8, requires p38MAPK, ATM, and NF-κB for transcriptional activation [5, 8, 14, 28, 29]. Additionally, p38MAPK regulates many SASP factors via post-transcriptional stabilization of their mRNA (6). However, not all SASP factors are regulated by these same pathways. For instance, while p38MAPK is an important regulator of the SASP, one study found that it regulated only 25 of 37 factors studied at the protein level while we previously reported that it regulates only 50 of 248 factors at the mRNA level in our model [5, 6].

One such factor is osteopontin (OPN), a pro-tumorigenic protein which has numerous physiological and pathological roles, including regulating bone turnover, cell adhesion and migration, and inflammation [30–33]. OPN is a secreted matrix protein that is upregulated in response to wounds, acts to recruit immune cells, can suppress apoptosis, and is upregulated and diagnostically relevant in a number of cancer types [32, 34–36]. OPN is also robustly upregulated in response to senescence. Previously we showed that senescent BJ skin fibroblasts lose the ability to promote preneoplastic cell growth when they are depleted of OPN. Furthermore, recombinant OPN induces preneoplastic cell growth in the absence of senescent cells [7, 37]. While the importance of senescent fibroblast-derived OPN is underscored by its ability to promote preneoplastic cell growth, the regulation of OPN in response to senescence is not understood. SASP regulators ATM and NF-κB are not required for OPN induction in response to senescence [38]. Other SASP regulators, such as C/EBPβ, have not been studied in conjunction with OPN [27, 29]. Indeed, there are no known regulators of OPN in response to senescence. Because of senescent-derived OPN’s ability to promote preneoplastic cell proliferation, it is important to understand how OPN is regulated in this context. Additionally, elucidating the regulation of OPN may provide insights into the regulation of other SASP factors that are regulated in a similar manner.

To identify regulators of OPN, we used an OPN promoter reporter to identify a senescence response element (SRE) that was required for activation of the OPN promoter in response to senescence. Using Transfac® to analyze the SRE for transcription factor binding motifs, we identified a number of putative regulators of OPN in senescence, including C/EBPβ and c-Myb. C/EBPβ is a transcription factor with important roles in senescence induction and is known to regulate IL-6, IL-8, and other SASP factors in response to senescence as well as in other contexts [39–41]. Likewise, it has been shown to regulate OPN in a lung cancer cell line [42]. c-Myb is a proto-oncogene important for hematopoietic development [43]. Additionally, c-Myb is a known regulator of OPN in hepatocellular carcinoma and melanoma models [32, 44]. While Myb family member b-Myb regulates proliferation and represses senescence, the Myb family transcription factors have distinct roles, and c-Myb has not been studied in mammalian cell senescence [45–48]. Interestingly, c-Myb and C/EBPβ can collaborate to activate transcription of a number of genes, including mim-1 and ChAT [49–51]. Therefore, we hypothesized that c-Myb and C/EBPβ regulate OPN and other SASP factors.

## RESULTS

### The OPN promoter contains a senescence response element

**Figure 1:**
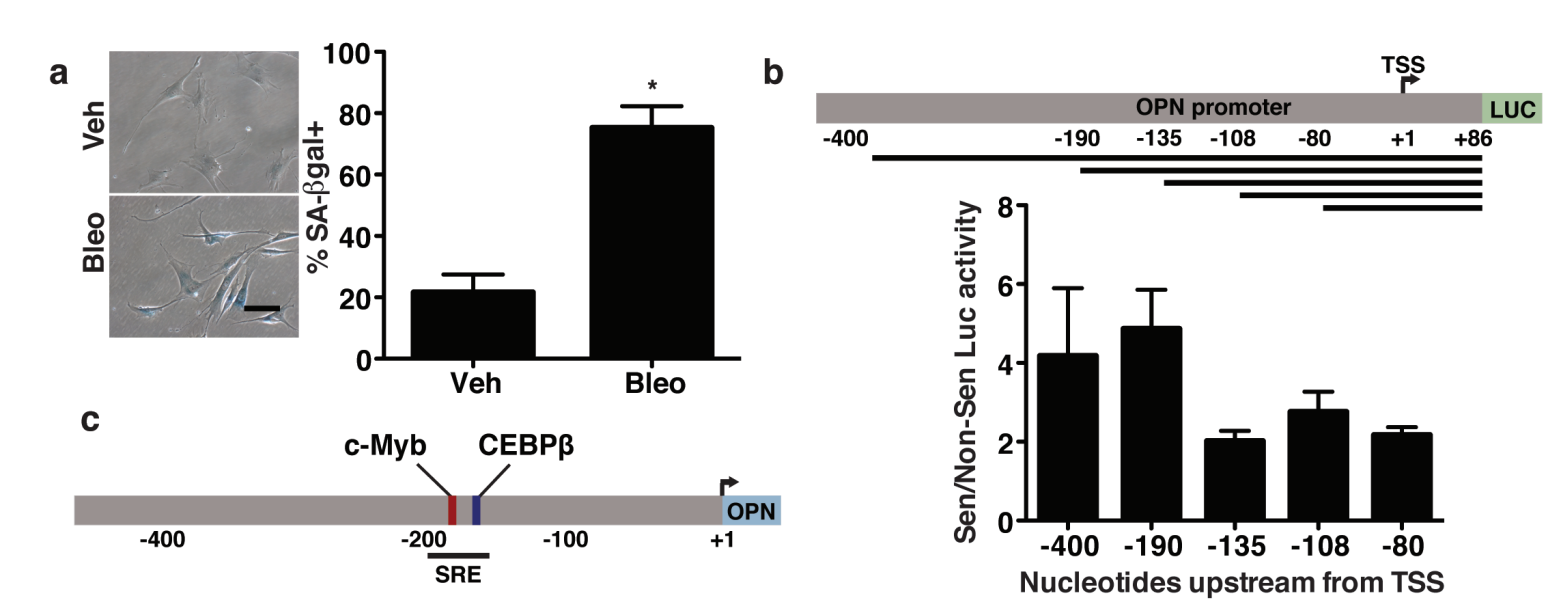
The senescence-responsive region of the OPN promoter contains c-Myb and C/EBPβ binding sites **a**. Treatment of BJ fibroblasts with bleomycin induces a significant increase in senescence as indicated by increased senescence-associated β-galactosidase staining, n=3, *p<0.05. Scale bar=100 μm **b**. Schematic of expression of luciferase reporter constructs that were driven by fragments of the OPN promoter spanning from nucleotide +86 to the indicated number of bases upstream from the transcription start site (TSS; top). BJ fibroblasts expressing the indicated promoter reporter constructs were treated with vehicle or bleomycin to induce senescence. Relative luciferase activity in senescent relative to non-senescent fibroblasts indicates a senescence-responsive element (SRE) between -135 and -190 bases upstream of the TSS (bottom), n=3. **c**. Schematic of OPN promoter. The SRE includes putative binding sites for the transcription factors c-Myb and C/EBPβ.

Given the pro-tumorigenic nature of OPN and its unique regulation among studied SASP factors, we sought to determine the mechanism of OPN regulation during senescence. To do so, we used promoter reporter constructs composed of regions of the OPN promoter driving transcription of luciferase to identify sequences in the OPN promoter required for transcriptional activation during senescence [52]. To carry out these analyses, BJ fibroblasts were transfected with the reporter constructs and induced to senesce by treatment with bleomycin for 24 hours. Four days after the start of bleomycin treatment, when cells displayed a senescent phenotype as demonstrated by a flattened morphology and staining positive for senescence-associated β-galactosidase (SA-βgal; Figure 1a), we assessed luciferase activity. We used regions of the OPN promoter spanning from the +86 nucleotide to the upstream site indicated (Figure 1b). While the +86 to -135 nucleotide region of the promoter had only ∼two-fold increase in luciferase activity in bleomycin treated cells relative to non-senescent cells, the region spanning from +86 to -190 nucleotides had 4.9-fold increased expression in senescent compared to non-senescent fibroblasts. The increased induction of expression observed in response to senescence when the -135 to -190 nucleotide region was present suggested that this region, which we termed the senescence response element (OPN-SRE), contains important senescence-associated transcription factor binding motifs (Figure 1c).

Transfac® analysis of the OPN-SRE promoter region revealed numerous putative binding sites for a variety of transcription factors, including HNF1, ZBTB16, HMGA1, SOX, FOXH1, C/EBPβ, and c-Myb. Preliminary data using shRNA to deplete cells of these transcription factors suggested that many were not required for OPN induction in response to senescence (data not shown). Therefore, we focused on the transcription factors C/EBPβ and c-Myb. C/EBPβ regulates the induction of numerous SASP factors including IL-6 and IL-8 in response to oncogene induced senescence [40]. In contrast, while c-Myb transcriptionally activates OPN in several epithelial cell models [32, 44], its roles in fibroblasts and in senescence are poorly studied. However, c-Myb and C/EBPβ can interact and have been shown to co-activate transcription of several genes in other settings [53–55]. Given these data, we examined C/EBPβ and c-Myb as possible regulators of OPN in response to senescence.

### C/EBPβ is required for robust OPN expression in response to senescence

**Figure 2:**
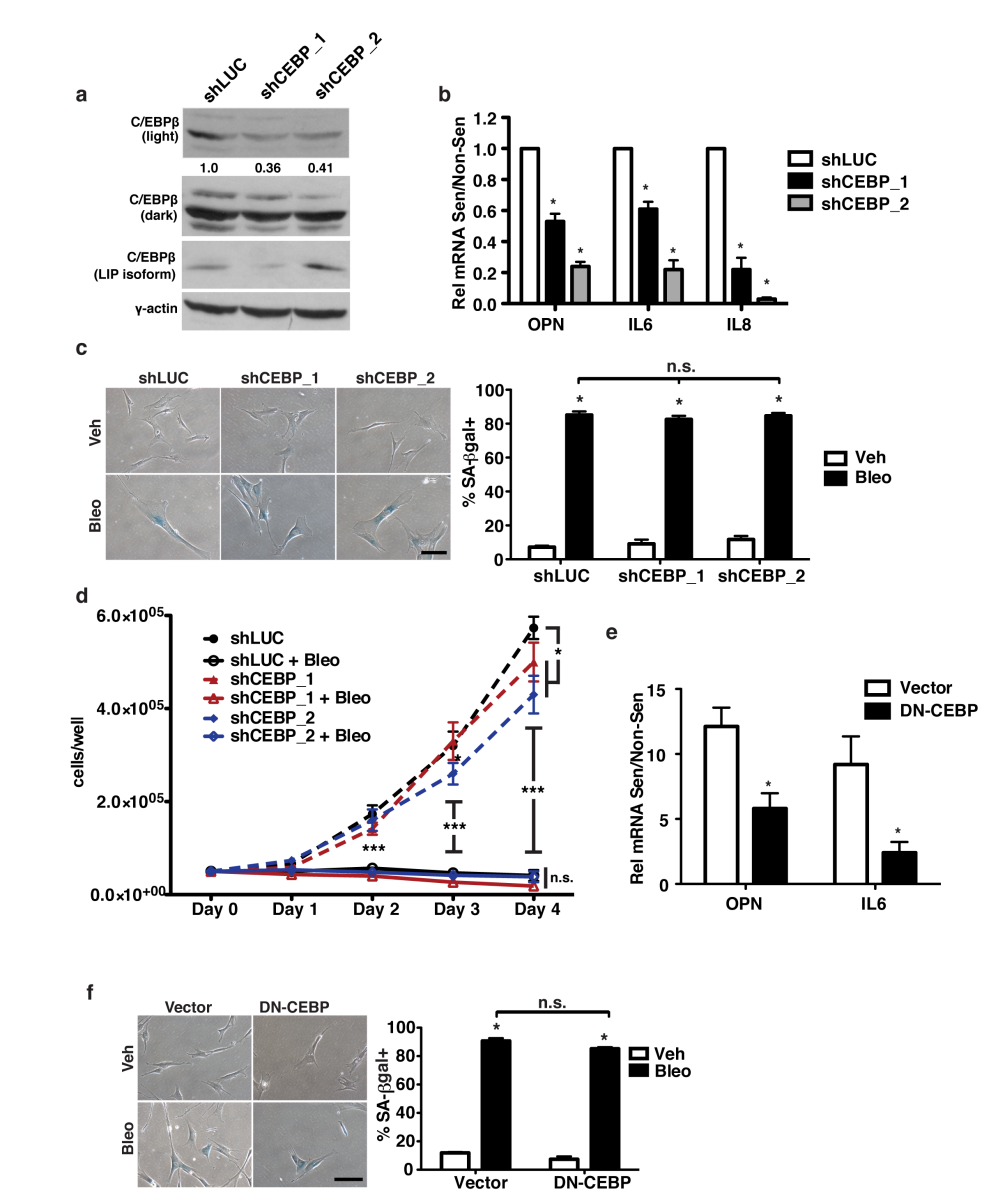
C/EBPβ is required for OPN induction in response to senescence **a**. C/EBPβ protein was measured in control or shCEBP-expressing cells via Western blot. shCEBP_1 had 64% reduced levels of activating C/EBPβ isoforms (LAP1&2) while shCEBP_2 had 59% reduced activating C/EBPβ isoforms, n=3. **b**. OPN, IL-6, and IL-8 mRNA expression were decreased in senescent shCEBP_1 and shCEBP_2 BJs relative to control (shLUC), n=3, *p<0.05. **c**. Senescence-associated β-galactosidase (SA-βgal) staining was used to measure senescence induction in BJ fibroblasts expressing one of two independent shRNAs targeting C/EBPβ (shCEBP_1, shCEBP_2) or a control hairpin (shLUC) and treated with bleomycin (Bleo, left). There is no significant difference in percent SA-βgal+ cells among any of the hairpins (right), n=3, n.s.=non-significant, *p<0.05. Scale bar=100 μm **d**. Cell proliferation over four days in non-senescent or bleomycin-treated fibroblasts was not affected by depletion of C/EBPβ, n=3, *p<0.05. **e**. Expression of OPN and IL-6 are significantly reduced in senescent cells expressing dominant negative C/EBPβ (DN-CEBP) relative to empty vector control, n=4, *p<0.05 **f**. SA-βgal staining indicates no change in senescence induction following bleomycin treatment in vector compared to DN-CEBP fibroblasts, n=3, n.s.=non-significant, *p<0.05, ***p<0.001. Scale bar=100 μm.

C/EBPβ regulates the induction of several SASP factors including IL-6 and IL-8 in response to oncogene induced senescence [40, 56] and has been implicated in control of OPN expression in other systems [42, 57, 58]. To test whether C/EBPβ was required for the induction of OPN in response to senescence, we depleted BJ fibroblasts of C/EBPβ using two independent shRNAs (Figure 2a). Upon the induction of senescence, C/EBPβ-depleted cells displayed reduced OPN expression relative to fibroblasts expressing a control short hairpin. Indeed, shC/EBPβ cells had 42% and 77% reduced OPN induction (shCEBP_1 and shCEBP_2, respectively) compared to shLUC expressing cells (Figure 2b). In agreement with previous studies [40], we found that C/EBPβ depletion also reduced IL-6 (39% and 78%, shCEBP_1 and shCEBP_2, respectively) and IL-8 (78% and 97%, shCEBP_1 and shCEBP_2, respectively) induction in response to senescence. Interestingly, depletion of C/EBPβ did not affect senescence induction in our system as measured by SA-βgal and cell growth measurement (Figure 2c-2d).

To confirm C/EBPβ’s role in regulating OPN, we inhibited C/EBPβ in BJ fibroblasts by stably expressing a dominant negative form of C/EBPβ, LIP. C/EBPβ has three isoforms: LAP1, LAP2, and LIP [59]. LAP1 and LAP2 are transcriptional activators while LIP, which contains the DNA binding domain but lacks the transactivation domain, is an inhibitory isoform that acts as a dominant negative to the activating isoforms. Compared to empty vector controls (Vector), OPN induction was reduced by 59% and IL-6 induction by 75% in LIP-expressing cells (DN-CEBP) in response to senescence (Figure 2e). Importantly, inhibition of C/EBPβ did not affect senescence induction as measured by SA-β-gal (Figure 2f). Thus, C/EBPβ is required for OPN, IL-6, and IL-8 induction in response to senescence, but depletion or inhibition does not prevent the induction of senescence in our system. C/EBPβ, therefore, represents a common factor of the previously distinct regulatory pathways of OPN and SASP factors such as IL-6 and IL-8.

### C/EBPβ binds to the OPN promoter

**Figure 3:**
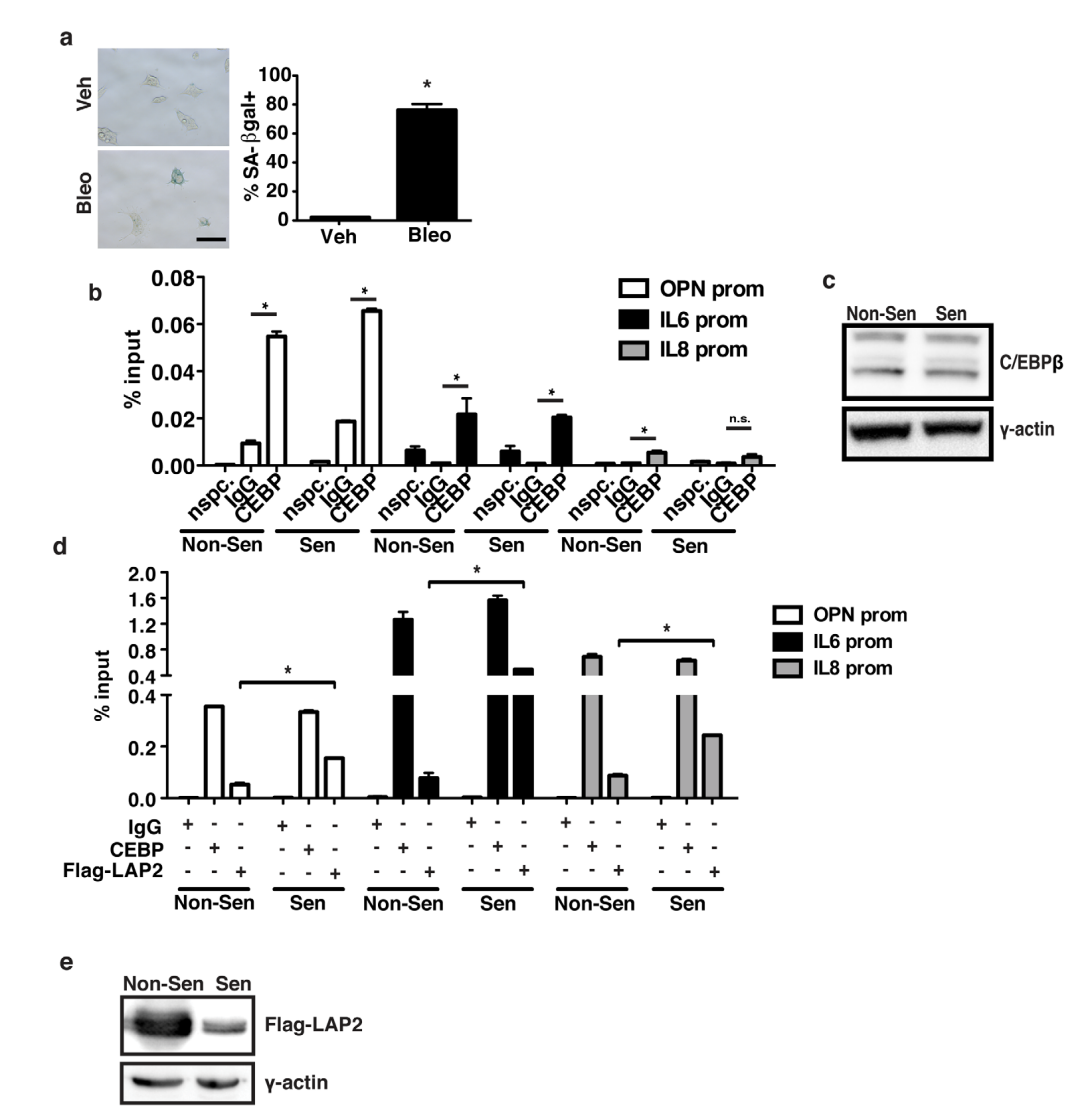
C/EBPβ isoform binds SASP promoters in senescent cells **a**. Treatment of 293T HEK cells with bleomycin induces a significant increase in senescence as indicated by increased senescence-associated β-galactosidase staining, n=3, *p<0.05. Scale bar=100 μm. **b**. Chromatin immunoprecipitation (ChIP) using a C/EBPβ antibody which recognizes all three C/EBPβ isoforms or a non-specific control (IgG) indicates that C/EBPβ significantly binds to the OPN and IL-6 promoters in both vehicle and bleomycin-treated 293Ts, and to the IL-8 promoter in vehicle treated cells, representative experiment, n=3, *p<0.05. “nspc.” indicates pulldown with C/EBPβ antibody at a distal site. **c**. Western blot using an anti-CEBPβ antibody indicating similar C/EBPβ expression in non-senescent and senescent ChIP treatments, n=1. **d**. ChIP in 293Ts transfected with a Flag-tagged activating C/EBPβ isoform (Flag-LAP2). An antibody recognizing all three C/EBPβ isoforms (CEBP) or an anti-Flag antibody was used to detect binding of the total C/EBPβ relative to exogenous Flag-LAP2 to the OPN, IL-6 and IL-8 promoters in vehicle (Non-Sen) and bleomycin-treated (Sen) 293Ts. While there was little change in total C/EBPβ bound to the OPN, IL-6, or IL-8 promoters, Flag-LAP2 binding to all three promoters was significantly increased in senescent cells, representative experiment, n=3, *p<0.05. **e**. Western blotting using an anti-Flag antibody indicated that Flag-LAP2 expression is significantly higher in non-senescent 293Ts than senescent 293Ts, n=3.

The promoters of OPN, IL-6 and IL-8 all contain C/EBPβ binding sites. To test whether C/EBPβ directly binds the OPN promoter, we used chromatin immunoprecipitation (ChIP) in non-senescent and bleomycin-treated 293T cells. In response to bleomycin, there was robust induction of senescence as measured by SA-βgal staining (Figure 3a). Because the upregulation of many SASP factors is transcription-dependent early after a senescence-inducing treatment but less dependent on transcription once senescence is fully established, we collected cells 48 h after the start of bleomycin treatment when transcription was robust [6]. Immunoprecipitation with an anti-C/EBPβ antibody that recognizes all three C/EBPβ isoforms revealed binding of C/EBPβ to the IL-6 and IL-8 promoters as has been previously shown (Figure 3b; [40]). In addition, there was significant binding to the OPN promoter in both non-senescent (0.05% input) and senescent (0.07% input) cells. Surprisingly, there was no significant difference in C/EBPβ binding relative to IgG in non-senescent compared to senescent cells. Likewise, there was little difference in C/EBPβ expression between the two conditions (Figure 3c). Interestingly, binding of exogenous Flag-tagged LAP2, an activating isoform of C/EBPβ, to the OPN, IL-6, and IL-8 promoters increased in response to senescence (Figure 3d). We observed this effect despite measuring greater Flag-LAP2 expression in non-senescent cells than in senescent cells (Figure 3e).

### c-Myb is required for robust OPN expression in senescent cells

Having established C/EBPβ as a regulator of OPN, we asked whether there were additional regulators in response to senescence. Thus, we returned to our promoter analysis to identify additional regulators of OPN. In addition to the C/EBPβ binding site, the SRE of the OPN promoter contains a putative c-Myb binding sequence. C-Myb is a proto-oncogene transcription factor but has never been implicated in mammalian senescence. Furthermore, while c-Myb is not well studied in fibroblasts, it has been shown to regulate fibrosis and many factors upregulated in fibrosis are also upregulated in senescence [60, 61], raising the possibility that it may play a role in regulating SASP factor expression. Importantly, c-Myb and C/EBPβ can interact and co-activate transcription in other contexts, suggesting they may act in a similar manner in response to senescence [53–55]. Interestingly, we also did not detect any change in c-Myb protein levels in response to senescence induction (data not shown).

**Figure 4:**
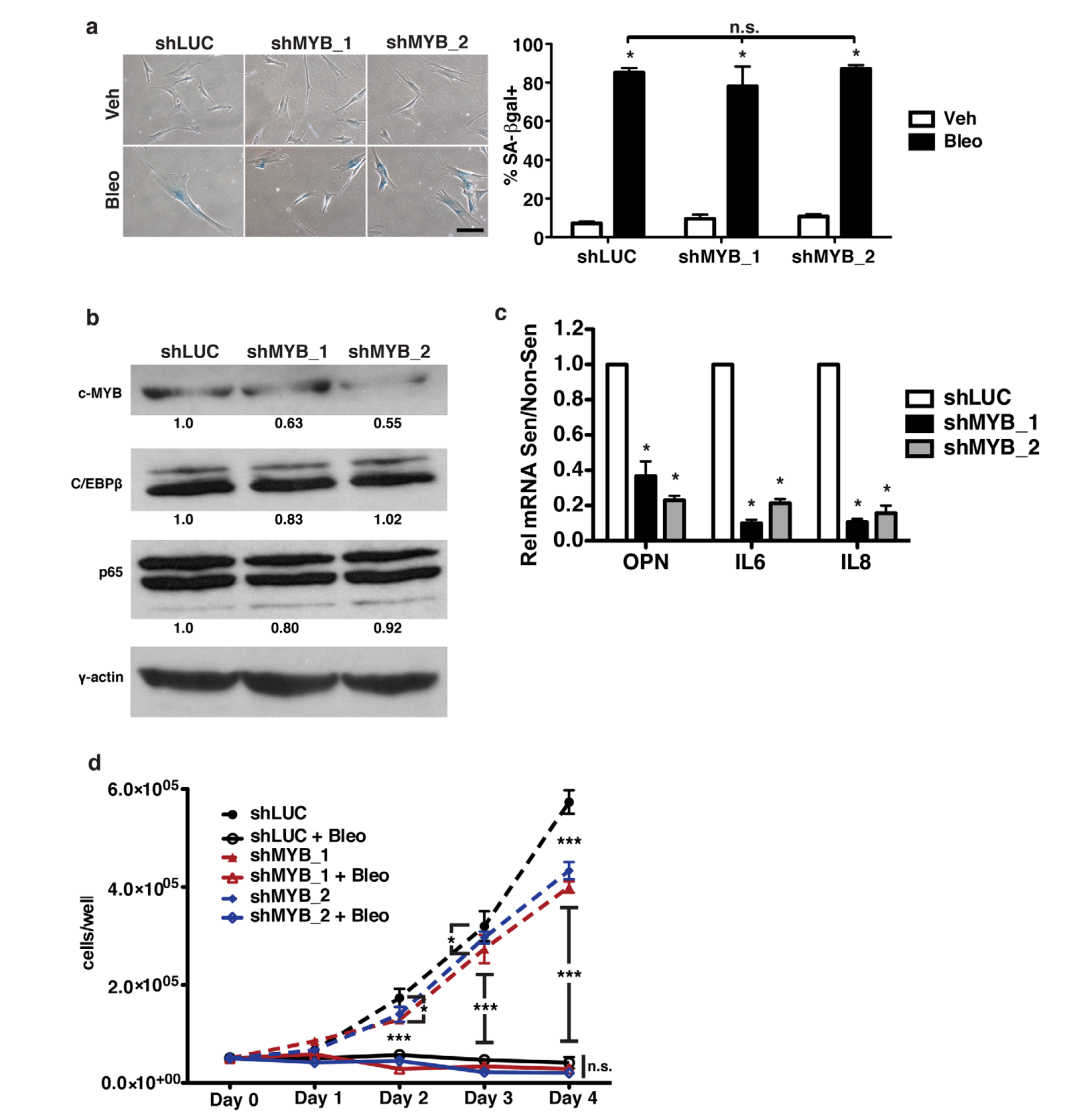
c-Myb regulates OPN, IL-6, and IL-8 in response to senescence **a**. Senescence-associated β-galactosidase (SA-βgal) staining was used to measure senescence induction in bleomycin-treated BJ fibroblasts expressing one of two independent shRNAs targeting c-Myb (shMYB_1, shMYB_2) or a control hairpin (shLUC; left). There is no significant difference in percent SA-βgal+ cells among any of the hairpins (right), n=3, n.s.=non-significant, *p<0.05. Scale bar=100 μm **b**. c-Myb, C/EBPβ and p65 protein levels were measured in control or shMYB-expressing cells via Western blot. shMYB_1 had 63% c-MYB while shMYB_2 had 55% c-Myb relative to control, n=3. **c**. OPN, IL-6, and IL-8 mRNA expression were decreased in senescent shMYB_1 and shMYB_2 BJs relative to control (shLUC), n=3, *p<0.05. **d**. Cell proliferation over four days in non-senescent or bleomycin-treated fibroblasts was not affected by depletion of c-Myb, n=3 *p<0.05, ***p<0.001.

To establish a role for c-Myb in the regulation of OPN during senescence, human fibroblasts were depleted of c-Myb using two independent short hairpins. Using bleomycin to induce senescence (Figure 4a), we measured OPN mRNA expression by qRT-PCR. Using two hairpins to deplete c-Myb, we observed a 37% and 45% (shMYB_1 and shMYB_2, respectively) reduction in c-Myb protein levels relative to γ-actin (Figure 4b), which resulted in a 64% and 77% decrease in OPN mRNA induction relative to shLUC control (Figure 4c). Importantly, depletion of c-Myb did not inhibit senescence-induction, as measured by SA-βgal and cell growth assays, indicating that c-Myb is necessary for OPN induction but not senescence induction (Figure 4a, 4d). In addition, depletion of c-Myb had little or no effect on C/EBPβ or NF-κB (p65) protein levels (Figure 4b), further indicating that c-Myb acts to transcriptionally regulate the SASP, not via indirect regulation of these transcription factors.

C-Myb has not previously been reported to regulate the SASP. Therefore, we asked whether c-Myb regulates other SASP factors in addition to OPN. Knockdown of c-Myb resulted in significantly reduced IL-6 (90% and 78%, shMYB_1 and shMYB_2, respectively) and IL-8 (89% and 84%, shMYB_1 and shMYB_2, respectively) mRNA expression in response to senescence (Figure 4c), indicating that c-Myb regulates multiple SASP factors and suggesting that it may broadly regulate C/EBPβ-dependent SASP factors.

### c-Myb regulates OPN via direct binding and activation of the OPN promoter

**Figure 5:**
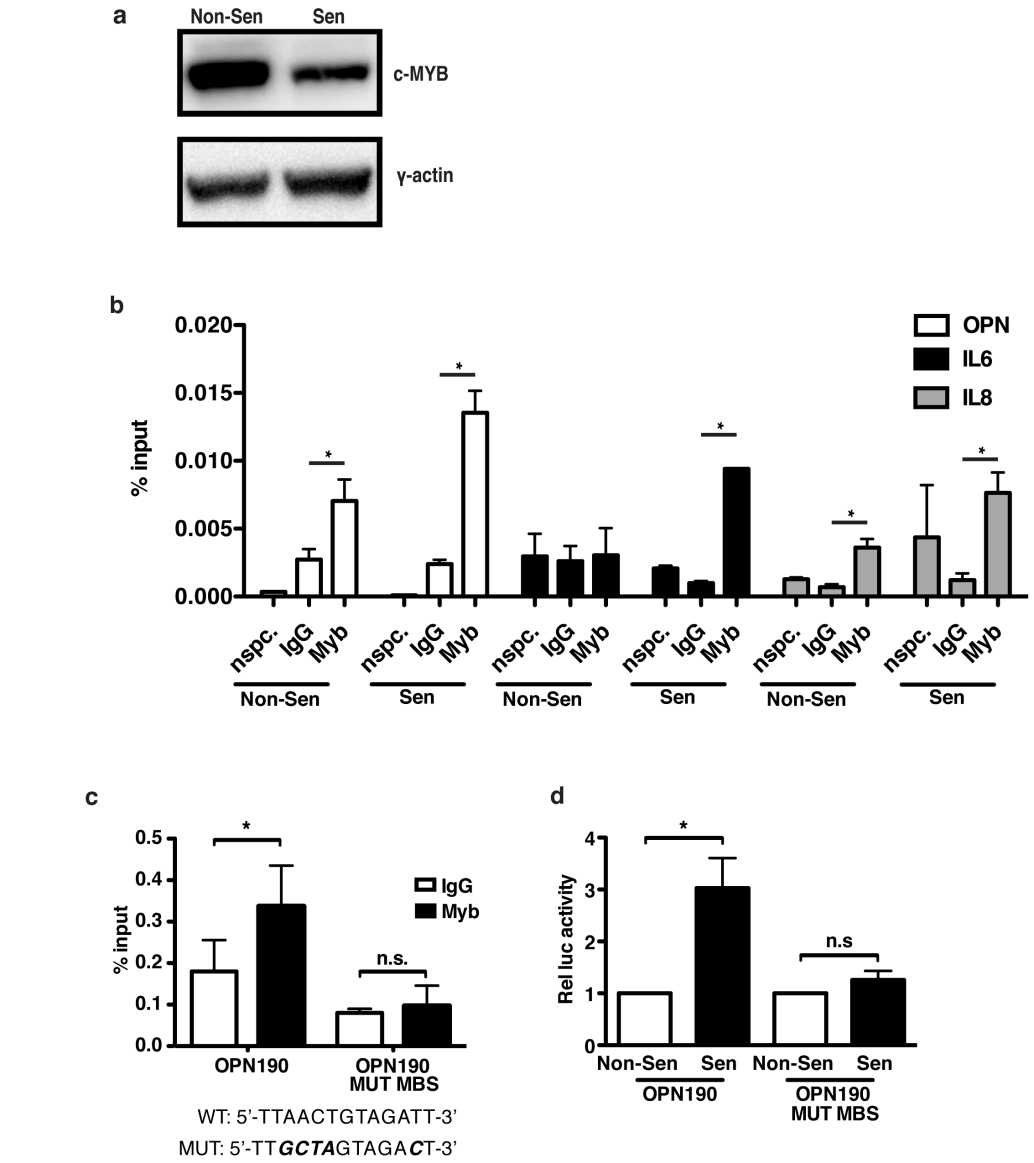
OPN induction in senescent cells requires c-Myb binding to the OPN promoter **a**. Western blot showing total c-Myb expression in the non-senescent and senescent cells used for ChIP. **b**. Chromatin immunoprecipitation was used to measure c-Myb binding to the OPN, IL-6, and IL-8 promoters in 293T cells expressing exogenous c-Myb cDNA. C-Myb significantly binds to all three endogenous promoters relative to IgG control, representative experiment, n=3, n.s.=non-significant, *p<0.05. “nspc.” indicates pulldown with c-Myb antibody at a distal site. **c**. c-Myb binds the OPN190 promoter reporter construct relative to IgG control. Mutation of the c-Myb binding site (mutations indicated below) eliminates binding to the OPN190 construct, n=3, n.s.=non-significant, *p<0.05. **d**. Luciferase activity is increased in senescent BJ fibroblasts expressing the WT OPN190 construct relative to non-senescent cells, but not in fibroblasts expressing the mutant c-Myb binding site OPN190 construct (OPN190MUT MBS), n=4, n.s.=non-significant, *p<0.05.

To test whether c-Myb directly regulates OPN transcription, we used chromatin immunoprecipitation (ChIP) in 293T cells ectopically expressing c-Myb (Figure 5a). Ectopic c-Myb was used because we were unable to consistently detect binding of endogenous c-Myb. ChIP analysis of c-Myb revealed that it is significantly bound to the endogenous OPN promoter relative to IgG control in both non-senescent (0.007 percent input Myb relative to 0.003 IgG) and senescent (0.014 percent input relative to 0.002 IgG; Figure 5b) cells. Additionally, c-Myb also bound the endogenous IL-6 and IL-8 promoters at similar levels, indicating that c-Myb directly regulates SASP factors other than a OPN. Further, in senescent cells c-Myb significantly bound transiently transfected WT OPN190-luciferase promoter reporter construct compared to IgG controls (0.34 c-Myb percent input relative to 0.18 IgG; Figure 5c). However, mutation of the putative c-Myb binding site on the OPN190 reporter (OPN190-MUT MBS) eliminated c-Myb binding (0.10 Myb percent input relative to 0.08 IgG), indicating that c-Myb binds specifically to this site. While WT OPN190 promoter activity is activated 4.3-fold following bleomycin treatment, OPN190-MUT MBS is not activated (1.3-fold; Figure 5d), indicating that c-Myb binding to the OPN promoter is required for the transcriptional induction of OPN following senescence induction. While this work was done with ectopic c-Myb, the ability to eliminate both c-Myb binding to the OPN promoter reporter construct and activation of the construct by mutating the c-Myb binding site suggests that endogenous c-Myb’s regulation of OPN may depend upon its ability to bind the endogenous promoter and that our inability to detect its presence on the promoter is due insufficient sensitivity in our assay.

### c-Myb and C/EBPβ regulate overlapping subsets of the SASP

**Figure 6:**
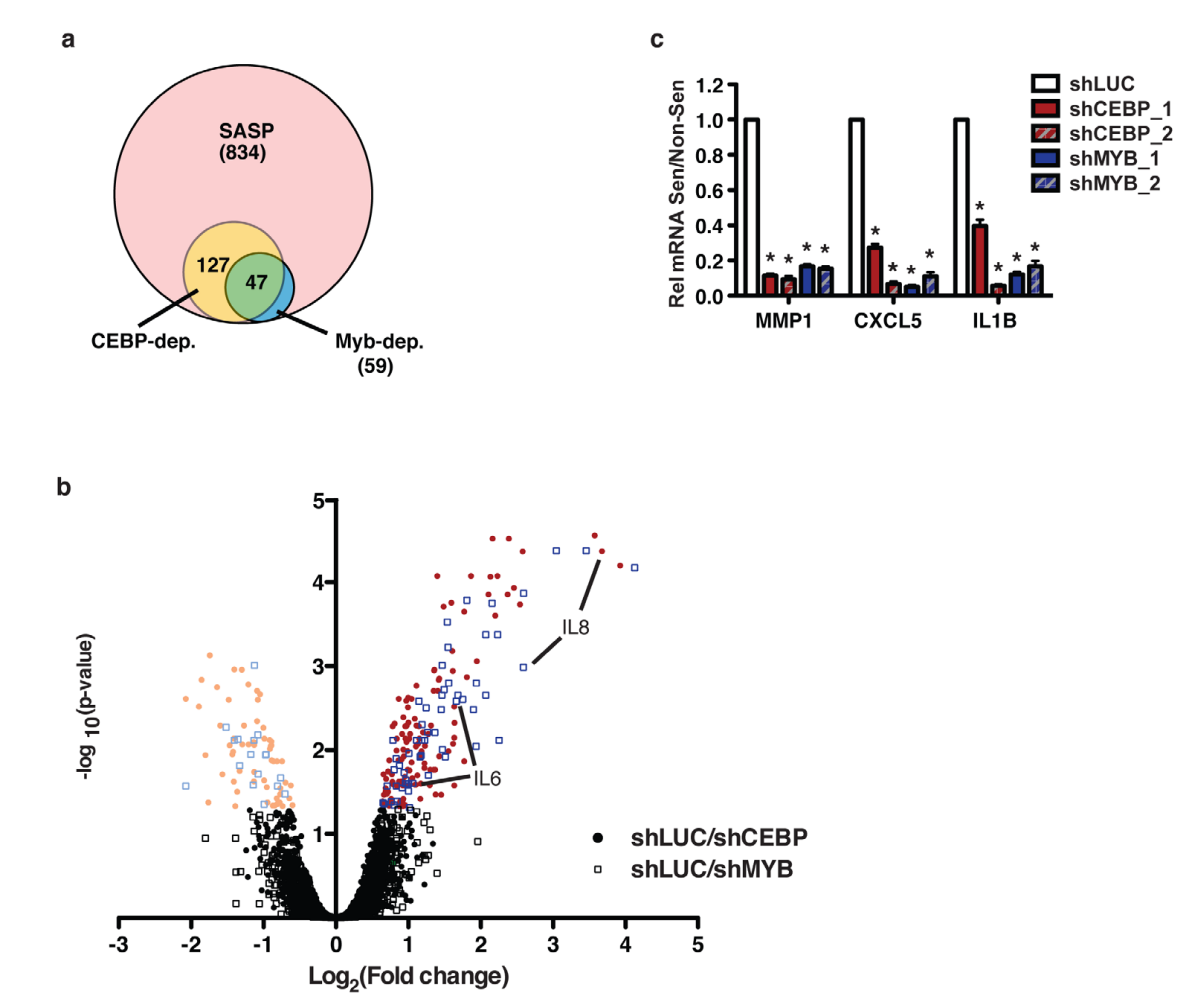
c-Myb and C/EBPβ regulate a subset of the SASP **a**. A microarray was performed to compare gene expression and induction in response to senescence among control, shCEBP_2 and shMYB_1 expressing fibroblasts. 834 genes were identified as SASP factors. Of these, 127 were solely C/EBPβ dependent, 12 were solely c-Myb dependent, and 47 were dependent on both transcription factors. Genes were considered dependent if their induction in response to senescence was significantly reduced in the experimental hairpin condition relative to control, *p*<0.05. **b**. The log2 fold change in induction (bleomycin over non-senescent) of shLUC relative to shCEBP_2 or shMYB_1 is plotted relative to the negative log10 of the p-value. Genes with significantly higher (bright colors, right) or lower (pale colors, left) induction in shLUC relative to shCEBP (circles) or shMYB (open boxes) are indicated. **c**. The C/EBPβ- and c-Myb-dependent SASP factors MMP1, CXLC5, and IL1B were validated using qRT-PCR. Depletion of C/EBPβ or c-Myb with two different hairpins each significantly reduced induction of these SASP factors.

While it has been shown that C/EBPβ regulates the SASP, c-Myb has not previously been implicated as a SASP regulator. To determine whether c-Myb regulates additional SASP factors beyond OPN, IL-6, and IL-8, we performed a microarray comparing transcript levels in non-senescent and senescent BJ fibroblasts expressing either a control short hairpin (shLUC) or a hairpin targeting either c-Myb or C/EBPβ (shMyb_1 or shCEBP_2, respectively). We restricted our analysis to 834 SASP genes, those which were significantly upregulated both by bleomycin-induced senescence and Ras-induced senescence (Supplementary Table 1). We compared the gene fold-upregulation in bleomycin-treated cells relative to untreated cells in the shLUC, shMYB_1, and shCEBP_2 groups. Comparing the fold-upregulation between groups, we found that 127/834 genes were C/EBPβ-dependent (Figure 6a, Supplementary Table 2). Importantly, 59/834 genes were c-Myb-dependent (Supplementary Table 3). Interestingly, 47/59 c-Myb-dependent genes were also C/EBPβ-dependent, suggesting c-Myb largely regulates C/EBPβ-dependent genes. We performed GO Term enrichment analysis on the SASP, C/EBPβ-dependent, and c-Myb-dependent gene sets. There were no significant enrichments among these gene sets relative to each other. However, the SASP was enriched for expected terms such as chemokine, cytokine, and extracellular matrix glycoprotein relative to the genome. Further, both C/EBPβ and c-Myb were similarly enriched for the terms chemokine, cytokine, and serine protease inhibitor.

In accordance with our qPCR findings, IL-6 and IL-8 are among the C/EBPβ- and c-Myb-dependent genes (Figure 6b). However, while OPN induction was reduced in shC/EBPβ and shMyb cells, this reduction was not significant. This difference was significant when measured by qPCR (Figure 4c), suggesting that the microarray data lacks sufficient power to find significance for genes with smaller changes. Therefore, our analysis likely underestimates the number of genes regulated by both C/EBPβ and c-Myb.

In addition to the genes we had already studied, we used qPCR to validate CXCL5, IL1β, and MMP1, three genes which were significantly dependent on both C/EBPβ and c-Myb in our microarray data. All three genes recapitulated the microarray results (Figure 6c). These data suggest that c-Myb is an important regulator of many C/EBPβ-dependent SASP genes in addition to OPN, IL-6, and IL-8.

### c-Myb and C/EBPβ knockdown inhibits preneoplastic cell growth promotion by senescent fibroblasts

Senescent fibroblasts promote the growth of neoplastic and preneoplastic epithelial cells in coculture and xenograft models via secretion of SASP factors [7, 37]. Depletion of OPN in senescent BJ skin fibroblasts is sufficient to eliminate the growth promotion that senescent BJ fibroblasts provide to HaCAT preneoplastic keratinocytes [7]. Because c-Myb and C/EBPβ regulate OPN and other SASP factors, we tested whether depletion of c-Myb and C/EBPβ would reduce the growth advantage provided by senescent cells using this same skin carcinoma coculture model.

**Figure 7:**
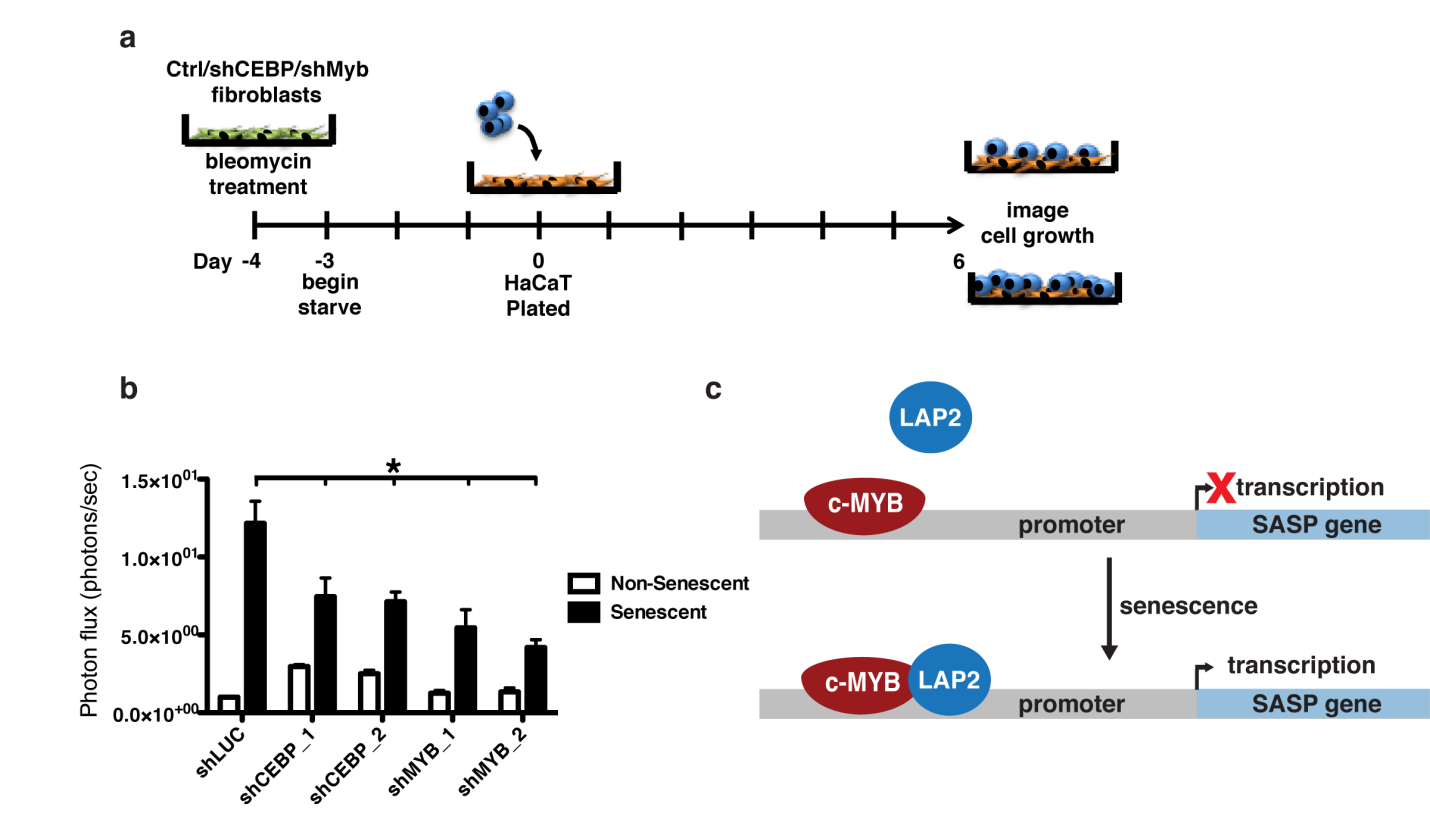
Depletion of c-Myb or C/EBPβ inhibits preneoplastic cell growth promotion by senescent fibroblasts **a**. HaCAT keratinocytes expressing CBR luciferase were cocultured with non-senescent or senescent BJs expressing either a control shRNA (shLUC) or shRNAs targeting C/EBPβ (shCEBP_1 and shCEBP_2) or c-Myb (shMYB_1 and shMYB_2). HaCAT proliferation was measured using bioluminescent imaging after six days of coculture, shown as fold growth when cocultured with senescent fibroblasts relative to non-senescent fibroblasts. **b**. Depletion of either C/EBPβ or c-Myb in senescent BJ fibroblasts significantly reduces the ability to promote HaCAT cell growth, n=3, *p<0.05. **c**. Schematic showing the proposed model of regulation of OPN and other SASP factors by c-Myb and C/EBPβ. In response to senescence, the activating isoform of C/EBPβ, LAP2, increases occupancy on the SASP promoter, inducing transcription.

HaCAT cells stably expressing click beetle red luciferase were plated on top of a confluent monolayer of 1.3x10^3^ non-senescent or senescent fibroblasts in serum-free media and allowed to grow for six days (Figure 7a). Recapitulating previous work, senescent fibroblasts dramatically increased HaCAT cell growth as measured by live cell imaging [7, 37]. However, this growth was significantly lower for HaCAT cells cocultured with either shC/EBPβ or shMyb expressing fibroblasts (Figure 7b), indicating the importance of C/EBPβ and c-Myb in regulating the SASP and its downstream pro-tumorigenic effects.

## DISCUSSION

The SASP plays important roles in wound healing and pathology, including the promotion of tumor development. Thus, understanding the complex regulation of the SASP will provide opportunities for therapeutic intervention. Many regulators of the SASP have been identified. However, it is clear that not all SASP factors are regulated by the same pathways. The SASP factor OPN is a potent pro-tumorigenic factor and is involved in numerous other physiological and pathological pathways such as bone turnover and the development of kidney stones [31, 62]. Previous work by our laboratory found that OPN is not dependent on the canonical SASP regulators ATM and NF-κB [38], illustrating that SASP factors are not subject to a single regulatory program.

We have identified C/EBPβ and c-Myb as critical regulators of the pro-tumorigenic SASP factor OPN. Depletion of C/EBPβ using shRNA and dominant negative inhibition significantly decreased OPN induction in response to senescence (Figure 2b, 2e). As has been previously reported, C/EBPβ is also required for the induction of IL-6 and IL-8 [40]. However, in contrast to that study which used an oncogene-induced senescence model, we did not observe a decrease in senescence induction in response to C/EBPβ depletion or inhibition (Figure 2a, 2d, 2f). While the cause of this difference is not clear, it may be due to differences in cell type, senescence-induction, or the level of C/EBPβ depletion. Nonetheless, the robust senescence induction observed, together with our ChIP data (Figure 3b, 3d), indicate that OPN is directly regulated by C/EBPβ and is not simply induced indirectly by the senescence program.

While C/EBPβ has previously been reported as an important SASP regulator, very little has been published about the mechanism of C/EBPβ activation of SASP genes. We observed that although there is no significant change in total C/EBPβ binding to the OPN, IL-6, or IL-8 promoters, binding of an exogenously expressed, activating form of C/EBPβ, Flag-LAP2, significantly increases in response to senescence (Figure 3b, Figure 3d). These data suggest that C/EBPβ regulation of SASP factor transcription may be more complex than simple binding, but require changes in specific isoform binding.

Our data establish that c-Myb is a novel regulator of components of the SASP. Depletion of c-Myb using shRNA significantly decreased the induction of OPN, IL-6, and IL-8 in response to senescence (Figure 4c). An additional 57 putative target factors were identified via microarray (Figure 6a), and three of these putative targets (MMP1, CXCL5, and IL1B) were validated with qRT-PCR (Figure 6c). The regulation of at least some of these genes may be direct, as exogenous c-Myb binds directly to the OPN, IL-6, and IL-8 promoters in both non-senescent and senescent cells (Figure 5b). Interestingly, despite lower ectopic c-Myb expression in senescent cells (Figure 5a), there is an increase in promoter occupancy in senescent cells relative to non-senescent, which, while non-significant, raises the possibility that c-Myb may increase binding to the promoters of some SASP factors in response to senescence. Using ChIP on exogenously transduced plasmids we were able to show that mutation of the c-Myb binding site on the OPN promoter disrupts this binding and abrogates promoter activation in response to bleomycin (Figure 5c-5d), suggesting that c-Myb binding is required for the activation of the promoter. Together these data indicate that c-Myb is critical for the induction of not only OPN, but a larger subset of the SASP. Although c-Myb has not been extensively studied in fibroblasts, one of its known roles is regulating fibrosis [60, 61]. Here we show that c-Myb regulates SASP factors, including matrix proteins OPN and MMP1, suggesting that c-Myb plays an important role in regulating the extracellular matrix in multiple physiological contexts.

C/EBPβ and c-Myb commonly act as co-activators of transcription [49–51]. Our data indicate that both transcription factors are required for the induction of OPN. In addition, via microarray analysis, we identified 47 additional SASP genes which are dependent on both C/EBPβ and c-Myb (Figure 6a). Only 12 c-Myb-dependent factors were not also C/EBPβ-dependent, indicating that c-Myb generally regulates C/EBPβ-dependent factors. We hypothesize that C/EBPβ and c-Myb interact to activate a cohort of SASP factors, but more work is needed to investigate whether these mechanisms studied in other contexts are also at play in senescent cells.

C/EBPβ and c-Myb are functionally important for the ability of senescent cells to promote the growth of pre-neoplastic HaCAT keratinocytes in a cocultures setting (Figure 7b). Depletion of either C/EBPβ or c-Myb significantly reduced the ability of senescent cells to promote HaCAT cell growth. Interestingly, depletion of C/EBPβ in non-senescent cells actually increased the growth promotion, but the mechanism of this increased growth is not known.

While OPN upregulation in senescence is independent of ATM and NF-κB, it does require C/EBPβ and c-Myb for expression. C/EBPβ and c-Myb also regulate IL-6, IL-8, and other NF-κB-dependent genes, suggesting that there are not simply distinct SASP master regulatory pathways, but multiple SASP regulators which act together and separately in a complex network to regulate the individual factors that are collectively the SASP. More work is needed to understand the interplay among the various regulatory pathways and which factors they regulate.

## MATERIALS AND METHODS

### Cell lines and treatments

Human foreskin BJ fibroblasts and 293T cells were cultured as previously described [7]. HaCAT preneoplastic keratinocyte cells stably expressing click beetle red (CBR) luciferase (HaCAT-CBR) and HEK 293T cells were grown in DMEM containing 10% heat-inactivated FBS and 1% penicillin/streptomycin (Sigma; [7]). All cells were cultured at 37°C in 5% carbon dioxide and 5% oxygen.

Cells were treated with 0.1 U/mL bleomycin sulfate (Sigma) for 24 hours. Cell pellets were collected 96 hours after the start of bleomycin treatment and RNA was isolated using TRI Reagent (Life Technologies) and Ambion RNA Isolation kit (ThermoFisher).

### Plasmids

OPN promoter luciferase constructs consisted of a fragment of the OPN promoter driving expression of luciferase in the pGL3 vector [52]. Fragments used were OPN80 (nucleotide [nt] -80 to nt +86), OPN108 (nt -108 to nt +86), OPN135 (nt -135 to nt +86), OPN190 (nt -190 to nt +86), and OPN400 (nt -400 to nt +86). The nt reported correspond to those upstream (-) or downstream (+) of the transcriptional start site. The OPN-LUC promoter constructs were a gift from the Paul C. Kuo Lab [52]. OPN190-LUC mutant c-Myb binding site was created using QuikChange II Site-Directed Mutagenesis (Agilent) and by following manufacturer’s protocol. The c-Myb binding site was changed from 5’-ttaactgtagatt-3’ to 5’-tt*gcta*gtagact-3’. pCMV-FLAG-LAP2 (Addgene plasmid #15738) and pBabe-puro LIP (Addgene plasmid #15713) were gifts from Joan Massague [63]. pCDNA3.1-Myb was a gift of Dr. Robert Rosenberg. pWZL hygro H-Ras V12 was a gift from Scott Lowe (Addgene plasmid # 18749, [10]). shMyb_2 (pSIREN-RetroQ-MYB-shRNA) was a gift from Judy Lieberman (Addgene plasmid # 25790; [64]). All other shRNA constructs were obtained from the Children’s Discovery Institute’s viral vector-based RNAi core at Washington University in St. Louis and were supplied in the pLKO.1-puro backbone. The sequences are as follows: shLUC (5’-TCACAGAATCGTCGTATGCAG-3’), shCEBP_1 (5’-CGACTTCCTCTCCGACCTCTT-3’), shCEBP_2 (5’-GCACAGCGACGAGTACAAGAT-3’), shMYB_2 (5’-CCAGATTGTAAATGCTCATTT-3’).

### SA-βgal

Senescence-associated-β-galactosidase staining was carried out as previously described [7].

### Growth assay

50,000 untreated or bleomycin-treated BJ fibroblasts expressing the indicated short hairpins were plated 96 hours after the start of treatment as previously described. Cell number was counted daily for four days using a hemocytometer. Significance was determined using a 2-way ANOVA with Bonferroni post-test.

### Western blot

Cell pellets were lysed in buffer containing 50 mM Tris pH 8.0, 5 mM EDTA, 0.5% NP-40, and 100 mM sodium chloride for 20 minutes at 4°C. Protein concentration was quantified using the Bradford Protein Assay (Bio-Rad). Membranes were blocked for one hour in 5% milk in TBS-T. The primary antibodies used were mouse monoclonal anti-FLAG M2 (Sigma; catalog number F1804) diluted 1:1000; rabbit polyclonal anti-C/EBPβ (Santa Cruz sc-150) diluted 1:2500; rabbit polyclonal anti-c-Myb rabbit polyclonal (Santa Cruz sc-517) diluted 1:250; and anti-γ-actin (Novus; catalog number NB600-533) diluted 1:5000. All secondary antibodies from the appropriate species were horseradish peroxidase–conjugated (The Jackson Laboratory) and diluted at 1:10,000. All antibodies were diluted in 2% BSA (Sigma-Aldrich) in TBS-T or 1% milk in TBS-T.

### Viral transduction

Viral transduction was performed as previously described [7]. All constructs were stably expressed using viral transduction unless otherwise noted.

### Luciferase reporter assay

BJ fibroblasts were transiently co-transfected with pGL3-Renilla and pGL3-OPN constructs using Lipofectamine 2000 (Thermo Fisher Scientific) and promoter activity was determined 48 hours later using Promega Dual Luciferase Reporter Assay (Promega) by following manufacturer’s protocol.

### Chromatin immunoprecipitation

Cells were transiently transfected with pcDNA-c-Myb WT (Myb), pGL3-OPN190 (OPN190), pGL3-OPN190 mutant c-Myb binding site (OPN190 Mut), or pCMV-Flag-LAP2 in 15 cm plates using 7 µg DNA and the TransIT® LT1 Reagent transfection system (Mirus). Cells were fixed 48 hours later using 1% formaldehyde in PBS for 20 minutes. Fixation was quenched with 125 mM glycine for 5 minutes with gentle rotation, cells were washed with PBS and collected by scraping and centrifugation at 200xg for 5 minutes at 4°C. Cells were lysed in 2 mL lysis buffer (1% SDS, 10 mM EDTA, 50 mM Tris pH 8.1) containing protease inhibitors (pepstatin, 1 μg/mL; aprotinin, 1 μg/mL; leupeptin, 1 μg/mL; PMSF, 100 μM) for 15 minutes. The lysate was sonicated at 50 Amps with 30 s on, 30 s off for 6 rounds to achieve DNA fragments approximately 200-500 bp in length as measured by electrophoresis. One mg protein was used for each immunoprecipitation. Lysate was diluted fivefold into ChIP dilution buffer (0.01% SDS, 1.1% Triton X-100, 1.2 mM EDTA, 16.7 mM Tris-HCl pH 8.1, 167 mM NaCl) and incubated at 4°C overnight with 5 μg appropriate antibody with vertical rotation. Antibodies used: anti-Myb (Santa Cruz sc-517); anti-C/EBPβ (Santa Cruz sc-150); Rabbit IgG (Cell Signaling 2729); anti-Flag M2 (Sigma F1804); mouse IgG1 (for Flag ChIPs, Cell Signaling 5415). Primers used: OPN TSS (Taqman assay # AJRR84Z), OPN190 (binds to the plasmid only; F:CTTTATGTTTTTGGCGTCTTCCA, R: CTAGCAAAATAGGCTGTCCC), IL-6 promoter (F: 5’-GCCATGCTAAAGGACGTCACA-3’, R: 5’-GGGCTGATTGGAAACCTTATTAAGA-3’), IL-8 promoter (F: 5’-AAGTGTGATGACTCAGGTTTGC-3’, R: 5’-GCACCCTCATCTTTTCATTATG- 3’), OPN distal (F: 5’-GTGGCTTCATGGAAACTCCCT-3’, R; 5’-GGACAACCGTGGGAAAACAA), IL-6 distal (F: 5’-CCATCCTGAGGGAAGAGGG-3’, R: 5’-CGTCGGCACCCAAGAATTT- 3’), IL-8 distal (F: 5’-TTTGGAGAGCACATAAAAACATC-3’, R: 5’-CAGCCAAAACTCCACAGTCA-3’).

### Quantitative PCR

cDNA synthesis and quantitative PCR was performed using manufacturer’s instructions (SYBR Green, Life Technologies and Taqman, Applied Biosystems). Primers used: GAPDH (F: GCATGGCCTTCGGTGTCC, R: AATGCCAGCCCCAGCGTCAAA), IL-6 (F: ACATCCTCGACGGCATCTCA, R: TCACCAGGCAAGTCTCCTCA), IL-8 (F: GCTCTGTGTGAAGGTGCAGT, R: TGCACCCAGTTTTCCTTGGG), OPN (F: TTGCAGCCTTCTCAGCCAA, R: AAGCAAATCACTGCAATTCTC), c-Myb (IDT PrimeTime® Std qPCR assay #Hs.PT.58.264008, Probe: 5’-56-FAM/CCTTCCGAC/ZEN/GCATTGTAGAATTCCAGT/3IABkFQ/-3’, F: 5’-CTCCTGCAGATAACCTTCCTG-3’, R: 5’-GCAGAAATCGCAAAGCTACTG-3’), C/EBPβ (Taqman assay # Hs00270923_s1), MMP1 (IDT PrimeTime® Std qPCR assay #Hs.PT.58.38692586, Probe: 5’-56-FAM/TCCGTGTAG/ZEN/CACATTCTGTCCCTG/3IABkFQ/-3’, F: 5’-GCCAAAGGAGCTGTAGATGTC -3’, R: 5’-GACAGAGATGAAGTCCGGTTT -3’), CXCL5 (IDT PrimeTime® std qPCR assay #Hs.PT.58.41058007.g, Probe: 5’-/56/FAM/CGGGGAGGG/ZEN/CAGGGAAGATG/3IABkFQ/-3’, F: 5’-GAACAGGCTTTACATTCAGACAG-3’, R: 5’-GGGTTAGAGGATTGCAGAAGA-3’), IL1β (IDT PrimeTime® std qPCR assay #Hs.PT.58.1518186, Probe: 5’-/56-FAM/AGAAGTACC/ZEN/TGAGCTCGCCAGTGA/3IABkFQ/-3’, F: 5’-GAACAAGTCATCCTCATTGCC-3’, R: 5’-CAGCCAATCTTCATTGCTCAAG-3’).

### Microarray

Microarray analysis was performed by the Genome Technology Access Center at Washington University. Cells expressing shLUC were senesced using bleomycin or Ras expression. Further analysis was restricted to genes that were either significantly up- or down-regulated in both bleomycin and Ras groups. Fold changes in bleomycin relative to untreated groups were then compared between the shLUC, shCEBP_2, and shMYB_1 groups. Two biological replicates for each group were analyzed. Statistical analysis was done using linear model fitting and the R package limma using an adjusted *p*-value<0.05 as the cutoff for significance [65,66]. GO Term Enrichment Analysis was performed using the Gene Ontology Consortium PANTHER software version 11.1 (released 2016-10-24) and the PANTHER Overrepresentation Test (released 2016-07-15). The PANTHER protein class annotation was used and our datasets were compared to the Homo Sapiens reference list with Bonferroni correction for multiple comparisons [67].

### Coculture

Coculture experiments were performed as previously described with the following modifications [7]. A total of 1.3 × 10^4^ fibroblasts were plated in black-walled 96-well plates (Fisher Scientific). Cells were incubated in starve medium (DMEM + 1% penicillin/streptomycin) for 3 days before the addition of HaCAT-CBR cells. HaCAT-CBR cells were cultured in starve medium for 24 hours before plating on fibroblasts. A total of 1.0 × 10^3^ HaCAT-CBR cells were plated on fibroblasts and incubated for six days. On day six, live-cell bioluminescence imaging was performed on an IVIS 50 (PerkinElmer; Living Image 4.3, 1 min exposure, bin8, FOV12cm, f/stop1, open filter). D-luciferin (150mg/ml; Gold Biotechnology, St. Louis, MO) was added to black-walled plates 10 min prior to imaging.

### Statistical analysis

Data is presented as the mean ± SEM. Student’s t-test was used to determine significance when comparing two groups. When comparing three or more groups, one-way ANOVA with Dunnett’s post-test was used, except where noted. In all cases, a *p*-value less than 0.05 was considered significant.

## SUPPLEMENTARY MATERIALS TABLES






