# Supplementary material for: c-Myb and C/EBPβ regulate OPN and other senescence-associated secretory phenotype factors
Source: Oncotarget. 2017 Dec 5;9(1):21–36. doi: 10.18632/oncotarget.22940 (PMC5787458; doi:10.18632/oncotarget.22940)
Supplement: Supplementary file 1 [file oncotarget-09-21-s001.pdf]

c-Myb and C/EBPβ regulate OPN and other senescence-associated secretory phenotype factors

SUPPLEMENTARY MATERIALS

For Supplementary Tables see in Supplementary Files.
