# Supplementary material for: c-Myb and C/EBPβ regulate OPN and other senescence-associated secretory phenotype factors
Source: Oncotarget. 2017 Dec 5;9(1):21–36. doi: 10.18632/oncotarget.22940 (PMC5787458; doi:10.18632/oncotarget.22940)
Supplement: Supplementary file 2 [file oncotarget-09-21-s002.docx]

CXCL5 59.6 28.5

CXCL8 26.8 27.6

SERPINB4 98.9 25.0

IL1B 22.1 14.2

CLDN1 14.2 13.2

IL24 6.5 11.0

IL13RA2 38.9 7.9

CXCL1 3.1 7.6

EREG 16.0 6.9

IL1A 29.4 6.6

GDF15 3.9 6.5

SERPINB2 9.3 6.2

C3 2.0 6.1

LOC105369848 20.4 6.1

MMP3 9.5 5.8

ESM1 9.0 5.7

CTSS 7.5 5.4

LOC541472 2.6 5.4

SLC16A6 40.9 5.2

RRAD 4.9 4.8

ITGA2 12.5 4.7

TFPI2 6.1 4.7

C15orf48 5.1 4.5

ACPP 29.8 4.4

KCTD4 14.9 4.4

MIR222 1.9 4.3

CSF3 8.2 4.2

IL6 1.9 4.1

SPP1 4.9 4.0

HIST1H2BG 5.5 3.9

C1QTNF1 1.4 3.9

AMPD3 6.6 3.9

TMEM158 6.6 3.8

LIF 4.4 3.8

TM4SF1 11.5 3.8

LINC01021 1.5 3.7

SLC22A4 2.7 3.7

KRTAP2-3 2.0 3.7

ULBP1 4.0 3.7

KRTAP3-1 2.9 3.6

ACER2 1.5 3.5

LINC01291 2.2 3.4

PRLR 2.1 3.3

ENC1 4.3 3.3

SAT1 5.1 3.3

PAPPA 1.5 3.3

TREM1 1.4 3.3

CXCL3 2.7 3.3

CCL20 1.9 3.3

CD24 1.5 3.3

STC1 5.8 3.2

CDCP1 12.4 3.2

LRRC15 7.5 3.2

PSTPIP2 1.9 3.2

P3H2 2.9 3.2

CSF2 21.5 3.2

NFKBIZ 3.7 3.1

CYFIP2 2.2 3.1

PI3 3.1 3.1

LOC105374003 43.9 3.0

C10orf55 17.5 3.0

THSD1 1.6 3.0

CES2 1.4 3.0

IFI30 1.7 3.0

PAG1 3.6 3.0

LCE1F 416.1 2.9

TNFAIP3 2.9 2.9

DUSP4 9.2 2.9

WDR63 2.0 2.9

STEAP1 5.2 2.9

MMP1 4.3 2.9

PTGS2 2.0 2.9

PTPN22 2.7 2.9

SEC11C 3.4 2.9

LRRN3 1.7 2.8

CHST7 2.6 2.8

LPXN 4.7 2.8

PLAU 15.0 2.8

DLGAP1-AS2 2.3 2.8

ERN1 3.3 2.8

COL10A1 36.4 2.7

IRAK3 1.6 2.7

DTNA 2.3 2.7

POU2F2 5.7 2.7

MAMDC2 1.5 2.7

HIST1H2BC 3.2 2.7

LOC105374171 4.7 2.7

RRM2B 2.2 2.7

IRAK2 4.6 2.7

EHF 7.8 2.7

VTRNA1-3 1.9 2.6

HIST1H4H 2.7 2.6

TSPAN13 7.2 2.6

TNFRSF10A 2.0 2.6

STAT4 1.9 2.6

APLP1 1.3 2.5

NEFM 4.0 2.5

HIST1H2BN 2.0 2.5

CDKN1A 1.4 2.5

AOX1 1.4 2.5

GADD45A 1.7 2.5

SOD2 1.7 2.5

PHLDA1 4.7 2.5

LOC105369808 2.3 2.5

AKR1B1 2.0 2.5

LOC105369893 29.2 2.4

HSD11B1 1.6 2.4

DUSP6 9.1 2.4

TMEM132A 2.4 2.4

PMAIP1 7.0 2.4

ANOS1 19.2 2.4

RAB27B 2.3 2.4

CYP3A7 2.4 2.4

LOC105376374 9.6 2.4

RNF152 4.7 2.3

SERPINB3 5.6 2.3

TIGAR 2.0 2.3

42797 2.6 2.3

SEMA3A 5.7 2.3

MLLT11 6.3 2.3

TMEM68 1.5 2.3

INA 3.0 2.3

FJX1 3.0 2.3

SHC4 2.0 2.3

EVI2A 1.4 2.2

IL11 6.0 2.2

PARM1 1.6 2.2

STEAP2 4.0 2.2

LCE2A 39.8 2.2

EPG5 1.9 2.2

ZC3H12C 4.1 2.2

DYRK3 2.2 2.2

ADRB2 1.8 2.2

NRG1 4.4 2.2

DHRS7 2.3 2.1

LOC105369340 2.0 2.1

POPDC3 5.9 2.1

GM2A 1.8 2.1

RHBDF2 2.5 2.1

HIST2H2BE 2.2 2.1

RNF19B 1.7 2.1

GK 2.9 2.1

MAP3K5 3.4 2.1

EPHA2 2.1 2.1

BCL2L1 2.0 2.1

ITGA6 4.6 2.1

PID1 3.1 2.1

LOC105376626 7.5 2.1

IER3 2.9 2.1

CASP3 3.6 2.1

ABL2 2.6 2.1

DGKA 1.4 2.1

PCDH9 4.2 2.1

FOLR3 1.8 2.1

GDNF 2.2 2.1

ARHGEF28 5.0 2.1

ANGPTL4 1.3 2.1

SMURF2 3.2 2.1

C8orf4 1.8 2.1

OR51A4 1.6 2.0

ZC3H12A 2.2 2.0

DLL4 4.6 2.0

RAP1GAP2 2.0 2.0

ATP6V0A1 2.7 2.0

FAM180A 1.8 2.0

LOC105374433 1.6 2.0

PLK3 2.5 2.0

SNORA14B 2.7 2.0

THEMIS2 3.0 2.0

HERC5 1.8 2.0

FBXL19-AS1 1.4 2.0

NPC1 2.0 2.0

DOCK5 1.9 2.0

NCEH1 2.0 2.0

LOC105376236 1.5 2.0

ATP13A3 2.4 2.0

SVIL 3.4 2.0

PIM2 2.0 2.0

HBEGF 1.4 2.0

ANPEP 3.8 2.0

PLD1 1.4 2.0

NT5E 3.4 2.0

ABCA1 3.2 2.0

TNFRSF10D 3.3 2.0

SUSD6 1.5 2.0

DUSP5 4.8 2.0

PGF 1.5 2.0

PLAT 2.1 2.0

OGFRL1 2.3 1.9

FBXO22-AS1 2.0 1.9

ODC1 3.5 1.9

TNFRSF10B 1.4 1.9

MIR4482 2.5 1.9

DNER 2.0 1.9

BCL2A1 4.4 1.9

NOMO1 1.5 1.9

MMP16 2.4 1.9

SNORD66 2.9 1.9

ABLIM3 3.6 1.9

LOC100507006 1.7 1.9

SLC39A14 1.7 1.9

TM7SF3 1.5 1.9

SLC9A1 2.3 1.9

ITGA3 2.5 1.9

LOC105373723 1.6 1.9

LAPTM5 2.0 1.9

SERPINB7 1.8 1.9

TAF13 3.0 1.9

SIPA1L3 3.3 1.9

ABTB2 1.4 1.9

PTCHD4 1.5 1.9

DAZL 7.1 1.9

METTL6 1.8 1.9

PRKX 1.6 1.8

MT1L 4.9 1.8

LOC105379676 1.9 1.8

CPEB4 1.9 1.8

LOC105376694 5.0 1.8

TP53I3 1.5 1.8

STYK1 2.0 1.8

C16orf52 2.2 1.8

LOC101926893 1.9 1.8

TMEM154 6.9 1.8

CCND1 2.1 1.8

PNP 3.4 1.8

MYDGF 1.4 1.8

TRIB1 3.4 1.8

FBXO22 1.5 1.8

HIPK2 1.9 1.8

EML2 1.7 1.8

CYB5R2 2.0 1.8

SQRDL 1.7 1.8

LRP8 3.0 1.8

LUCAT1 1.7 1.8

GXYLT1 1.4 1.8

IGF2R 2.5 1.8

TPCN1 1.7 1.8

LOC105379695 2.3 1.8

SGTB 1.7 1.8

ERO1B 1.6 1.8

RASSF8 2.4 1.8

SLC8A1-AS1 2.0 1.8

HSPH1 2.0 1.8

CYB5R1 1.5 1.8

FBXO32 1.9 1.8

NEFL 9.2 1.8

KYNU 4.0 1.8

ATG4A 2.2 1.8

EDA2R 1.3 1.8

RETSAT 1.4 1.8

AIM1 2.0 1.8

TMEM38B 2.8 1.7

CYLD 1.7 1.7

LOC105369568 3.9 1.7

MIR146A 9.4 1.7

RPSAP52 1.6 1.7

SLC20A1 4.6 1.7

NMNAT2 1.6 1.7

PLAUR 2.4 1.7

MIR4451 2.2 1.7

INHBA 6.1 1.7

ARHGAP22 3.6 1.7

PLA2G4C 2.0 1.7

HAGH 1.3 1.7

TNIP1 1.7 1.7

GRAMD1B 1.9 1.7

EMC7 1.5 1.7

MMP12 13.1 1.7

ADGRE2 3.5 1.7

LOC105374745 3.8 1.7

DCBLD2 5.3 1.7

MAFF 2.0 1.7

HAS2 3.3 1.7

GSTO1 2.4 1.7

LINC01002 2.0 1.7

WDFY2 2.1 1.7

TSSC2 1.6 1.7

RND3 1.3 1.7

CPED1 2.3 1.7

MYOCD 1.6 1.7

SLC11A2 1.3 1.7

PLCB4 1.4 1.7

LAMC2 2.9 1.7

ADAM23 1.6 1.7

ABCA13 1.5 1.7

ASB5 3.3 1.7

POMGNT1 1.7 1.7

C9orf72 2.4 1.7

MYO6 1.4 1.7

SRA1 1.9 1.7

PROCR 2.1 1.7

CDIP1 1.5 1.7

DPP4 1.5 1.7

SERPINB10 1.4 1.7

CD274 6.9 1.7

AGTRAP 1.3 1.7

DCUN1D3 2.0 1.7

SESN2 1.4 1.7

MAP7 1.4 1.7

LOC101929470 1.8 1.7

NEDD4L 1.8 1.7

NFKBIA 1.4 1.7

C17orf89 1.3 1.7

PGPEP1 1.5 1.7

GPR183 3.5 1.7

PLEK2 15.7 1.7

ORMDL2 1.8 1.6

RAB3B 1.4 1.6

CITED4 4.0 1.6

DNAJB9 1.6 1.6

EVC 1.3 1.6

RRS1 1.7 1.6

GLA 2.6 1.6

LOC644135 2.2 1.6

UXS1 1.5 1.6

HK2 1.7 1.6

UCN2 3.7 1.6

CDA 1.7 1.6

WTAPP1 2.7 1.6

NOMO2 1.4 1.6

STK4 1.6 1.6

CABYR 1.4 1.6

YRDC 2.3 1.6

MTHFD2L 1.9 1.6

ETV1 4.3 1.6

MAP4K3 1.5 1.6

ATP2B1 2.5 1.6

PTP4A1 1.6 1.6

TNFRSF21 16.8 1.6

MICA 2.0 1.6

LOC101927121 1.7 1.6

DAGLB 1.4 1.6

NOMO3 1.4 1.6

PSMD2 2.3 1.6

DYNC1H1 2.0 1.6

ZNF468 1.3 1.6

KCTD1 1.3 1.6

SLC31A2 1.6 1.6

SRXN1 1.8 1.6

SLC4A7 1.4 1.6

ZCCHC6 2.1 1.6

RALA 3.4 1.6

E2F7 10.3 1.6

KIF21A 1.8 1.6

ELK3 1.6 1.6

MRPL39 1.5 1.6

UHRF1BP1L 1.6 1.6

NSF 1.6 1.6

MAP2K3 2.0 1.6

AEN 1.5 1.6

EPT1 2.1 1.6

OSGIN2 2.4 1.6

GPR4 6.7 1.6

TMEM63B 1.3 1.6

MYCT1 1.7 1.6

FEZ1 1.6 1.6

OSTM1 1.7 1.6

ETV4 3.1 1.6

PNPO 1.5 1.6

HERC4 1.4 1.6

ZNF267 1.6 1.6

HMGA2 2.5 1.6

WDR66 1.6 1.6

EAF1 1.8 1.6

SDC1 1.5 1.6

NOG 3.3 1.6

PSME4 1.8 1.6

C2orf81 2.2 1.6

NFKB2 1.6 1.6

TOP1 2.2 1.6

ITPRIP 2.6 1.6

ZPR1 2.1 1.6

LOC105377023 1.3 1.6

CHMP5 1.8 1.6

FCRLB 1.6 1.6

PPP1R15A 1.8 1.6

LOC101928820 2.1 1.6

LOC105369844 3.3 1.6

RABGGTA 1.4 1.6

PDGFC 1.4 1.6

GSAP 2.4 1.6

WDR43 1.4 1.6

MCTP1 7.7 1.5

ZNF432 1.4 1.5

ADIRF 1.7 1.5

KIF3B 1.6 1.5

FAM214B 2.1 1.5

UCHL3 2.0 1.5

EIF5A2 1.4 1.5

SOWAHC 1.6 1.5

ITPR3 1.7 1.5

BCAP31 1.6 1.5

RPLP0P2 12.1 1.5

SRPX2 1.6 1.5

C6orf1 1.3 1.5

MCC 1.5 1.5

HYOU1 2.0 1.5

GLRX 2.0 1.5

PPP3CC 1.4 1.5

PI4K2A 1.6 1.5

MYO5A 2.5 1.5

LINC00589 1.5 1.5

CREM 1.6 1.5

MAFK 1.5 1.5

FXR2 1.7 1.5

TNFAIP2 1.3 1.5

PAK2 1.4 1.5

ELL 1.9 1.5

ANKRD31 1.5 1.5

LOC105369313 2.8 1.5

LINC01204 2.1 1.5

FOSL1 1.7 1.5

LOC101928461 1.9 1.5

STEAP3 5.0 1.5

SCARB1 2.1 1.5

PARD6B 2.2 1.5

CORO2A 1.6 1.5

SLC35G2 1.5 1.5

TCEB3 1.5 1.5

STX3 1.5 1.5

DGKE 2.1 1.5

ARID3A 1.7 1.5

TRMT6 2.3 1.5

DNAJB11 1.4 1.5

MKLN1-AS 1.3 1.5

ATP6AP1 1.2 1.5

DDA1 2.0 1.5

NDUFAB1 2.0 1.5

GSS 1.9 1.5

DNAJC3 1.3 1.5

EGFR 1.4 1.5

GFM2 1.9 1.5

42798 1.3 1.5

AK5 1.9 1.5

LOC105369204 1.5 1.5

UBA6 2.1 1.5

NCLN 1.7 1.5

CLCA4 1.4 1.5

POLR3A 2.3 1.5

HSPA13 1.5 1.5

UBASH3B 1.6 1.5

ETNK1 1.9 1.5

FUCA2 1.3 1.5

DOCK4 12.8 1.5

NAV3 3.6 1.5

SPRY2 5.1 1.5

RIPK2 2.1 1.5

CNST 1.4 1.5

LURAP1L 1.3 1.5

PPFIBP1 1.5 1.5

ITPR2 2.0 1.5

PTPN1 1.9 1.5

UBE2M 1.9 1.5

ZNF276 1.5 1.5

FAM210B 3.5 1.5

NKX3-1 1.3 1.5

EDEM3 1.9 1.5

NR1D1 1.5 1.5

PWARSN 2.2 1.5

ABHD5 1.7 1.5

MYO10 2.6 1.5

PEX19 1.2 1.5

LOC100505622 1.5 1.5

MAP1A 1.8 1.5

ABHD3 1.7 1.5

PPP2R1B 1.5 1.5

NFKBIB 1.7 1.5

ATF3 1.4 1.5

BHMT2 2.9 1.5

GPR3 2.8 1.5

SCAMP3 1.3 1.5

LOC105376382 5.9 1.5

NEK10 1.6 1.5

LOC105379272 1.6 1.5

NEU1 1.7 1.5

SQSTM1 1.5 1.5

MAPKBP1 1.4 1.5

GDF11 1.3 1.5

SLFN5 1.6 1.5

LIG4 1.6 1.5

SMOX 1.7 1.5

PITPNC1 4.2 1.5

TLDC1 1.4 1.5

C15orf54 2.6 1.5

NCR3LG1 1.4 1.5

TNFAIP1 1.2 1.5

NIPA1 1.3 1.5

PRMT5 1.5 1.5

UHMK1 1.7 1.5

SATB2 2.6 1.5

DAP3 1.6 1.5

C3orf52 2.1 1.5

FLJ42627 1.4 1.5

METTL8 1.4 1.5

PDIA4 1.7 1.5

TBC1D9 1.7 1.5

DEDD2 1.4 1.5

FHOD3 2.5 1.5

G6PC 1.3 1.5

PFN2 1.3 1.5

ZNF622 2.1 1.5

C18orf8 1.4 1.5

TAB3 1.5 1.5

MAFG 1.6 1.5

ASB1 1.6 1.5

FAM214A 1.5 1.4

PNO1 2.0 1.4

ARFGEF2 1.4 1.4

MXD1 1.5 1.4

SLC22A1 1.4 1.4

TSPYL1 1.6 1.4

MSC 2.1 1.4

TOR1AIP2 1.4 1.4

PIP4K2C 1.5 1.4

MSI2 2.3 1.4

MFSD2A 1.6 1.4

PTPRF 1.3 1.4

COQ10B 2.1 1.4

PSMC5 1.5 1.4

CHMP1B 1.6 1.4

PSMC4 1.8 1.4

NFKBIE 1.3 1.4

MYEF2 1.4 1.4

FRMD5 1.7 1.4

MORN4 1.5 1.4

BVES 2.6 1.4

LOC101928225 1.5 1.4

PAQR5 1.5 1.4

NFE2L1 1.3 1.4

LOC105373813 1.4 1.4

WIPI1 1.5 1.4

PTRH2 1.9 1.4

TUSC2 1.5 1.4

KLHL32 1.4 1.4

CEP104 1.3 1.4

CCBE1 2.4 1.4

GGT3P 1.6 1.4

TERF2IP 1.6 1.4

SLC37A2 2.5 1.4

ASCC3 1.4 1.4

SEC23B 2.2 1.4

ELOVL4 2.0 1.4

MCL1 2.0 1.4

HIVEP2 1.3 1.4

CMTM4 1.4 1.4

MTOR 1.8 1.4

ZNF121 1.6 1.4

ACSL4 1.5 1.4

ADGRA3 1.6 1.4

RGAG4 1.6 1.4

FIBCD1 1.9 1.4

ATG2A 1.5 1.4

PDLIM4 1.9 1.4

MIR2909 1.5 1.4

TMEM131 1.8 1.4

PPIF 3.4 1.4

EMC1 1.4 1.4

ASAP2 2.0 1.4

GTF2F2 1.5 1.4

PSMD14 2.0 1.4

LOC105372190 2.0 1.4

SUCO 1.6 1.4

PKIA 6.4 1.4

SLC39A2 1.5 1.4

LAMA1 1.5 1.4

MMP14 2.0 1.4

LOC105374556 2.7 1.4

UNC13B 1.6 1.4

SENP5 1.6 1.4

LRP10 1.2 1.4

GABARAPL2 1.7 1.4

HMGXB3 2.5 1.4

URB1-AS1 1.5 1.4

TRIM25 1.5 1.4

CEP170B 1.2 1.4

SREK1IP1 1.5 1.4

SPRY4 4.3 1.4

TRIM23 1.3 1.4

PLEKHB2 2.1 1.4

EMP1 1.9 1.4

SLC35D1 1.4 1.4

SPATA17 1.6 1.4

SERINC2 2.7 1.4

UBXN8 1.5 1.4

SEC61A2 1.7 1.4

FADS1 1.7 1.4

AMN1 2.3 1.4

UST 2.1 1.4

IGDCC4 1.6 1.4

ECE1 1.6 1.4

FMN1 2.0 1.4

SMTN 1.5 1.4

NRIP3 4.9 1.4

DENND2A 2.7 1.4

DKK2 2.5 1.4

GALNT15 1.7 1.4

SNORD116-12 1.4 1.4

IL4R 1.6 1.4

LARP4 1.4 1.4

SBNO1 1.7 1.4

EDEM1 2.4 1.4

HMGA1 2.0 1.4

PSMD3 1.6 1.4

ERRFI1 4.6 1.4

DPP9 1.9 1.4

NLRP1 1.7 1.4

FMNL2 3.8 1.4

GTPBP4 1.9 1.4

TANC1 1.3 1.4

PIKFYVE 1.5 1.4

BTBD9 1.3 1.4

G0S2 3.1 1.4

SDF2L1 1.9 1.4

CCNH 1.3 1.4

DBNDD1 1.7 1.4

HS3ST3B1 2.3 1.4

SAMD8 1.8 1.4

UBALD2 2.2 1.4

HTT 2.1 1.4

ATP1B3 1.4 1.4

CLDN12 1.3 1.4

STXBP1 2.0 1.4

OXSR1 1.6 1.4

RNF181 1.4 1.4

GDPD1 2.5 1.4

EFTUD1 1.6 1.4

FGFR1OP 1.4 1.4

HSPA5 1.7 1.4

ZFAND2A 1.5 1.4

SDE2 1.9 1.4

KLHL21 1.3 1.4

QSOX2 1.8 1.4

IDS 1.5 1.4

WNK4 1.4 1.4

ZMYND8 1.8 1.4

TMEM8A 1.4 1.4

SECISBP2 1.4 1.4

MAP4K4 1.5 1.4

PPTC7 1.6 1.4

ITPKC 1.3 1.4

PISD 1.4 1.4

TFRC 1.6 1.4

SELPLG 1.3 1.4

TMCO1 1.4 1.4

WWC3 1.4 1.4

ZDHHC5 1.3 1.4

ATP6V1E1 1.6 1.4

CRADD 2.1 1.4

TOMM34 2.4 1.4

TMEM33 2.1 1.4

MTF1 1.4 1.4

GCC2 1.2 1.4

ANKRD52 1.7 1.4

LINC00884 1.2 1.4

PLCXD1 2.5 1.4

BAZ1A 1.6 1.4

CD109 1.3 1.4

GFPT1 1.7 1.4

NEDD4 1.4 1.4

GSG1 1.4 1.4

UAP1 1.4 1.4

LYST 1.8 1.4

FKBP4 2.0 1.4

BBS7 1.5 1.4

PIEZO1 1.3 1.4

RAB3IP 1.3 1.4

FBXO28 2.2 1.4

JARID2 1.8 1.4

RICTOR 1.5 1.4

AVL9 1.8 1.4

AUP1 1.4 1.4

TMEM2 1.8 1.4

MT2A 1.5 1.4

ARSG 1.8 1.4

NXPE3 1.3 1.4

ISOC1 3.1 1.4

PRKCD 1.6 1.4

RUSC2 1.4 1.4

BTN2A2 1.9 1.4

TSPAN14 2.9 1.4

GTF2B 1.5 1.4

HDAC9 1.5 1.4

VEPH1 3.0 1.4

JADE2 1.3 1.4

METTL13 1.3 1.4

DNAJC5 1.4 1.4

SLC3A2 1.5 1.4

RCL1 1.5 1.4

SLC17A5 1.4 1.4

UFD1L 1.6 1.3

CCL5 1.4 1.3

ORAOV1 1.7 1.3

BAZ2A 1.4 1.3

SLC33A1 1.7 1.3

RPRD1A 1.7 1.3

CHMP4C 1.4 1.3

BNC1 6.7 1.3

UGGT1 1.4 1.3

MAST4 1.4 1.3

CCND2 1.4 1.3

SLC2A6 2.0 1.3

TSTA3 1.2 1.3

ERLEC1 1.3 1.3

CD44 1.7 1.3

YIPF6 1.4 1.3

STYXL1 1.5 1.3

CUBN 1.9 1.3

KDSR 1.2 1.3

SMURF1 1.5 1.3

SRP54 1.6 1.3

PWAR5 2.6 1.3

ORAI1 1.5 1.3

SPTY2D1 1.5 1.3

COX7A2 1.5 1.3

TFAP2C 2.0 1.3

CDC37 1.6 1.3

CISD1 1.6 1.3

TRIM37 1.4 1.3

FAM91A1 1.7 1.3

HIST1H2AC 1.3 1.3

TOR4A 1.4 1.3

SERP1 1.3 1.3

AFF1 1.5 1.3

DESI1 1.8 1.3

PFKFB3 2.0 1.3

PLOD2 1.5 1.3

ARHGAP18 2.0 1.3

KLHL18 1.7 1.3

AKIRIN1 1.8 1.3

EIF1AY 1.6 1.3

EPB41L4B 1.5 1.3

PSMB7 1.5 1.3

HSPA9 1.7 1.3

TMED5 1.5 1.3

FAM96B 1.7 1.3

KIAA1217 1.3 1.3

TMED9 1.3 1.3

WSB2 1.3 1.3

ACO1 1.5 1.3

PTCHD3 1.4 1.3

ZBTB21 2.0 1.3

ATP6V1D 1.6 1.3

LOC105370145 1.3 1.3

PINK1 1.5 1.3

SLC4A4 2.8 1.3

IPPK 1.5 1.3

FAM129B 1.4 1.3

XPC 1.4 1.3

H2AFJ 1.3 1.3

ATP2C1 1.3 1.3

LHFPL2 1.8 1.3

ABHD2 1.5 1.3

CLCN3 1.4 1.3

BAK1 1.6 1.3

SMCR8 1.5 1.3

MOXD1 1.6 1.3

GSK3B 1.4 1.3

MBOAT7 1.3 1.3

CPNE3 2.3 1.3

LGALSL 1.5 1.3

CSRNP1 2.4 1.3

PRDM4 1.3 1.3

FLJ32255 1.4 1.3

TXNRD1 2.3 1.3

CANT1 1.4 1.3

SPIRE1 1.7 1.3

SFXN4 1.3 1.3

MCFD2 1.2 1.3

SEC14L2 1.3 1.3

PDHX 1.4 1.3

LRRC36 1.3 1.3

UBR4 1.5 1.3

SYNRG 1.4 1.3

UROD 1.4 1.3

SYNE1 1.2 1.3

LOC102723721 1.4 1.3

PCNXL4 1.4 1.3

ACO2 1.4 1.3

SIK3 1.3 1.3

PHYH 1.4 1.3

PIN1 1.3 1.3

GAPVD1 1.6 1.3

MYBBP1A 1.6 1.3

RPTOR 1.3 1.3

PLAA 1.4 1.3

KCNMA1 1.7 1.3

PSEN2 1.3 1.3

DUSP1 1.4 1.3

CPEB1 1.9 1.3

TMEM120B 1.4 1.3

SERPINE1 2.1 1.3

KLC1 1.4 1.3

COQ6 1.3 1.3

TMEM57 1.4 1.3

ALG2 1.4 1.3

TMBIM1 1.2 1.3

GRPEL1 2.0 1.3

MLLT4 1.4 1.3

ATP6V1H 1.9 1.3

C2CD2L 1.8 1.3

PHLDA2 3.4 1.3

PVR 2.0 1.3

SLC19A2 1.4 1.3

CES1P1 1.3 1.3

PITRM1 1.3 1.3

DYRK1B 1.3 1.3

QPCTL 2.0 1.3

UEVLD 1.3 1.3

VEGFC 2.5 1.3

B4GALT7 1.2 1.3

VCP 1.4 1.3

BECN1 1.2 1.3

FXYD5 1.4 1.3

EIF4E 1.8 1.3

LOC727896 1.7 1.3

USP36 1.4 1.3

MICALL1 1.2 1.3

RASA2 1.3 1.3

ADIPOR1 1.3 1.3

SLAIN2 1.5 1.3

SLC22A5 1.2 1.3

CCT3 1.7 1.3

P4HA2 1.3 1.3

TRAF3IP2 1.3 1.3

TRMT1 1.7 1.3

DIS3 1.5 1.3

UCK2 1.7 1.3

ALAS1 1.6 1.3

CCNDBP1 1.2 1.3

ICOSLG 1.3 1.2

PTAR1 1.7 1.2

SLC30A7 1.7 1.2

GHITM 1.5 1.2

NRDC 1.4 1.2
