# Supplementary material for: c-Myb and C/EBPβ regulate OPN and other senescence-associated secretory phenotype factors
Source: Oncotarget. 2017 Dec 5;9(1):21–36. doi: 10.18632/oncotarget.22940 (PMC5787458; doi:10.18632/oncotarget.22940)
Supplement: Supplementary file 3 [file oncotarget-09-21-s003.docx]

CXCL5

EREG

SERPINB2

CXCL8

IL13RA2

SERPINB4

CXCL1

MMP1

LPXN

TMEM158

IL24

TM4SF1

ESM1

LOC105376382

IL1B

STC1

TFPI2

LOC105376374

CTSS

ACPP

C3

PTGS2

CDCP1

CSF2

SLC22A4

MMP3

CYB5R2

PI3

RNF152

NFKBIZ

DENND2A

SOD2

FAM180A

CSF3

ANLN

C2orf81

LCE2A

DTNA

KIF2C

HMGA1

MAP3K5

AKR1B1

ITGA6

ODC1

LINC01291

PLAT

CPED1

SMURF2

TREM1

HAS2

PHLDA1

FJX1

CXCL3

CEP55

PLAU

ANPEP

TNFAIP3

DUSP6

FHOD3

CDK1

IRAK3

PID1

ITGA2

SEMA3A

VEPH1

LIF

IL1A

DLL4

AIM1

APCDD1

SPC24

PLK1

NT5E

FOSL1

CLDN1

AMPD3

CCL20

LOC105374171

RGCC

ERRFI1

SLC8A1-AS1

LOC105369848

CDKN3

OGFRL1

NCEH1

EMP1

COL10A1

SHCBP1

TOP2A

ELK3

SHC4

TMEM132A

PAQR5

ARHGAP18

RPSAP52

IL6

C10orf55

AOX1

IL11

PRLR

TRIM55

PLEK2

SERPINB3

ETV4

ANOS1

RASSF8

PDIA4

SLC39A14

WDFY2

HSD11B1

OR51A4

RIPK2

LCE1F

LMNB1

MARCH3

AK5

APLP1

UAP1

INA

DUSP4

SLC16A6

SLC35G2

NEDD4L

CASP3

PDLIM4

ENC1

FCRLB
